# Minocycline-associated rimmed vacuolar myopathy in a patient with rheumatoid arthritis

**DOI:** 10.1186/1471-2377-12-140

**Published:** 2012-11-21

**Authors:** Kota Bokuda, Keizo Sugaya, Shunichiro Tamura, Kazuhito Miyamoto, Shiro Matsubara, Takashi Komori

**Affiliations:** 1 Department of Neurology, Tokyo Metropolitan Neurological Hospital, 2-6-1 Musashidai, Fuchu, Tokyo, 183-0042, Japan; 2 Department of Laboratory Medicine and Pathology, Tokyo Metropolitan Neurological Hospital, 2-6-1 Musashidai, Fuchu, Tokyo, 183-0042, Japan

**Keywords:** Minocycline, Autophagic vacuole, Rimmed vacuolar myopathy, Minocycline-induced pigmentation, Rheumatoid arthritis

## Abstract

**Background:**

The autophagic vacuolar myopathies (AVM) are a group of inherited myopathies defined by the presence of autophagic vacuoles in pathological muscle specimens. AVM can be categorized into three groups: acid maltase deficiency, myopathies characterized by autophagic vacuoles with unique sarcolemmal features, and rimmed vacuolar myopathies (RVM). While the pathogeneses of these conditions are still being elucidated, some drugs (e.g., chloroquine, its analog, hydroxychloroquine, and colchicine) can also cause AVM. Minocycline is a disease-modifying anti-rheumatic drug that may be used in the treatment of rheumatoid arthritis (RA). Here, we describe the first case of minocycline-associated AVM with rimmed vacuole formation.

**Case presentation:**

A 75-year-old woman suffering from RA has been continuously treated with minocycline (200 mg/day) for the past 7 years. During this time, she developed a myopathy that predominantly affected her lower limbs. Histological studies of biopsied muscle revealed scattered atrophic myofibers with rimmed vacuoles that contained pigment granules. Histochemical staining revealed that the pigment comprised both iron and melanin, which is consistent with type II minocycline-induced cutaneous pigmentation. Under electron microscopy, autophagic vacuoles were consistently observed in association with numerous collections of pigment granules.

**Conclusions:**

This is the first report of minocycline-induced pigmentation in skeletal muscle. The strong association between autophagic vacuoles and the accumulation of minocycline-induced pigments suggest that long-term minocycline treatment induced pigment accumulation, leading to elevation of autophagic activity and RVM. It might also be possible that minocycline directly activated autophagy, as the observed pigments are known to form complexes containing minocycline and/or its metabolites. As long-term minocycline treatment is expected to be used more widely in the future, we must draw attention to this adverse effect.

## Background

Autophagy, which is a lysosomal degradation pathway that is essential for cell survival [1], plays a key role in the pathogenesis of several inherited myopathies; these include autophagic vacuolar myopathies (AVM) [2] as well as the drug-induced AVM caused by chloroquine, hydroxychloroquine and colchicine [3,4]. Both inhibition and activation of autophagy can lead to the appearance of autophagic vacuoles in pathological muscle specimens [1,2]. In addition to the best characterized AVM, Pompe disease, two other categories of AVM have recently emerged. One is Danon disease and its related disorders, which are characterized by autophagic vacuoles with unique sarcolemmal features [5]. The other is typified by the presence of rimmed vacuoles (vacuoles within myofibers that are lined with blue granular material following hematoxylin-eosin staining or with red material following modified Gomori trichrome staining); electron microscopy has revealed that these are actually clusters of autophagic vacuoles [5].

Minocycline is a semi-synthetic derivative of tetracycline that functions as a broad-spectrum antimicrobial agent and has proven to be a moderately effective disease-modifying anti-rheumatic drug in the treatment of rheumatoid arthritis (RA) [6]. The drug is generally well tolerated, but long-term use of minocycline (100 to 200 mg/day), especially in the management of acne vulgaris or RA, can be associated with a number of side effects. The most common adverse drug reactions are photosensitivity and esophagitis, while the rarer adverse events include severe allergic reactions, such as Stevens-Johnson syndrome, systemic lupus erythematosus, serum sickness, autoimmune hepatitis, vasculitis and dermatomyositis [7,8]. Long-term use of minocycline can also cause pigmentation, which is a well-recognized adverse effect that affects the skin and other organs [9]. To our knowledge, this is the first report of rimmed vacuolar myopathy (RVM) associated with long-term minocycline treatment, wherein cluster of autophagic vacuoles are associated with the accumulation of minocycline-induced pigmentation.

## Case presentation

### Clinical findings

A 75-year-old woman was admitted to our hospital due to a 2-year history of gradually progressive gait disturbance. She had suffered from RA since the age of 25. Because of an exacerbation of this disease, she started taking prednisolone (5 mg/day) and sulfasalazine at 67 years, and had been continuously treated with minocycline (200 mg/day) since the age of 68. She noticed a gradual increase in blue-black skin pigmentation on both legs at 72 years. Thereafter, she began to have difficulty walking, and would frequently catch the tip of her foot on the ground. No exposure to other drugs known to cause pigmentary changes was recorded.

**Figure 1:**
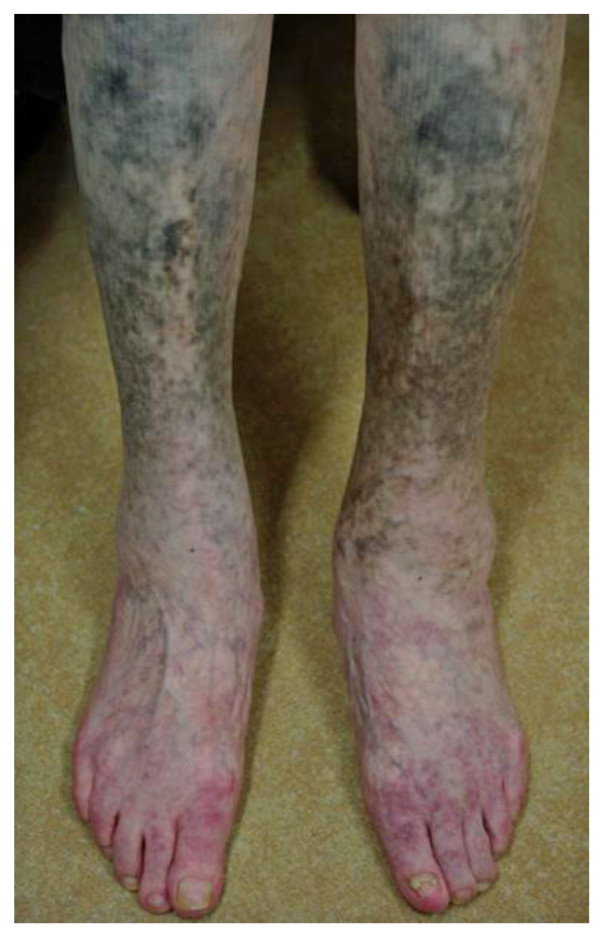
**Skin hyperpigmentation of the patient.** Blue-black pigmentation related to minocycline therapy was observed on the distal parts of the patient’s legs.

Examination of the patient revealed blue-black pigmentation related to minocycline therapy on the distal parts of the legs (Figure 1). The muscle strength was normal in the upper limbs and trunk, but she was unable to perform toe- or heel-walking. There was mild symmetrical weakness and atrophy in the lower limb muscles, but no wide-based gait. Strength testing was performed using Medical Research Council grades, and the results were as follows: hip flexion 4+/5, hip extension 4+/5, knee extension 4/5, knee flexion 4-/5, ankle dorsiflexion 4-/5, and ankle plantar flexion 4-/5. The tendon reflexes were normal except for the absence of ankle jerks. A stocking distribution decrease in appreciation of superficial pain was identified in both legs. Vibration sensation was decreased in the toes and ankles. Laboratory tests, including creatine kinase levels, were normal except for the tests related to RA. Chest roentgenogram and electrocardiogram showed no abnormalities. A nerve conduction study demonstrated no detectable abnormalities except for low amplitude in the bilateral sural nerve action potentials. Needle electromyography showed low amplitude and short duration motor-unit potentials with early recruitment in the quadriceps femoris, biceps femoris and tibialis anterior muscles. No abnormal spontaneous activity was seen in any of the muscles. Skeletal muscle CT revealed diffuse muscular atrophy of the lower extremities. Because the patient was suspected of having myopathy and sensory neuropathy, peroneus muscle and sural nerve biopsies were performed after informed consent was obtained.

### Histology

**Figure 2:**
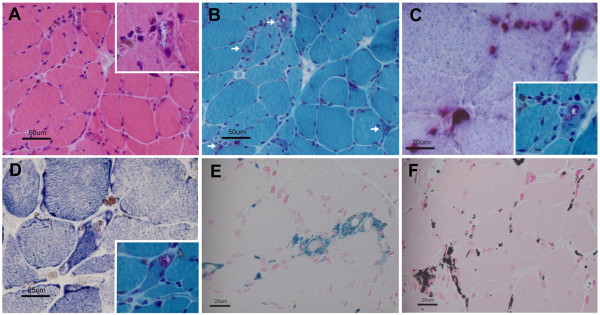
**Light microscopy of muscle biopsy. A**: Modest variability in myofiber diameter with scattered angular atrophic fibers (hematoxylin-eosin staining). Rimmed vacuole is shown at higher magnification (insert). **B**: Atrophic fibers with rimmed vacuoles (arrow, modified Gomori trichrome staining). **C**: Granular pigment-containing histiocytes in endomysial areas and rimmed vacuoles showing high acid phosphatase activity (compared to the rimmed vacuole on modified Gomori trichrome staining [insert]). **D**: NADH-tetrazolium reductase reaction shows dark-brown depositions in myofibers and vacuoles (compared to the rimmed vacuole on modified Gomori trichrome staining [insert]). **E**: Histiocytic pigment stained bright blue with Prussian blue. **F**: Histiocytic pigment stained black on a Masson Fontana preparation.

The biopsied specimens were snap-frozen in isopentane-liquid nitrogen, and cryosections were stained with a standard battery of histological and histochemical reactions. Paraffin-embedded tissue sections were also stained with Prussian blue stain (for iron), Masson Fontana preparation (for melanin) and hydrogen peroxide melanin bleach. Histopathological studies of the biopsied muscle revealed modest variability in myofiber diameter with scattered angular atrophic fibers (Figure 2A). There were a considerable number of atrophic fibers with rimmed vacuoles (Figure 2B), and multiple collections of granular pigment-containing histiocytes in the endomysial and perimysial perivascular areas. The granules and rimmed vacuoles showed high acid phosphatase activity (Figure 2C). The pigmentation was mainly found outside the myofibers, but the NADH-tetrazolium reductase reaction readily identified dark-brown depositions in some fibers. Almost all of the rimmed vacuoles contained granular depositions (Figure 2D). No necrotic or regenerating fibers were present. There was no inflammatory infiltrate, except for histiocytes containing pigment that stained bright blue with the Prussian blue (Figure 2E). This histiocytic pigment stained black on a Masson Fontana preparation (Figure 2F), bleached in response to potassium permanganate, and showed no fluoresce under ultraviolet light, indicating that it was composed of iron and melanin.

**Figure 3:**
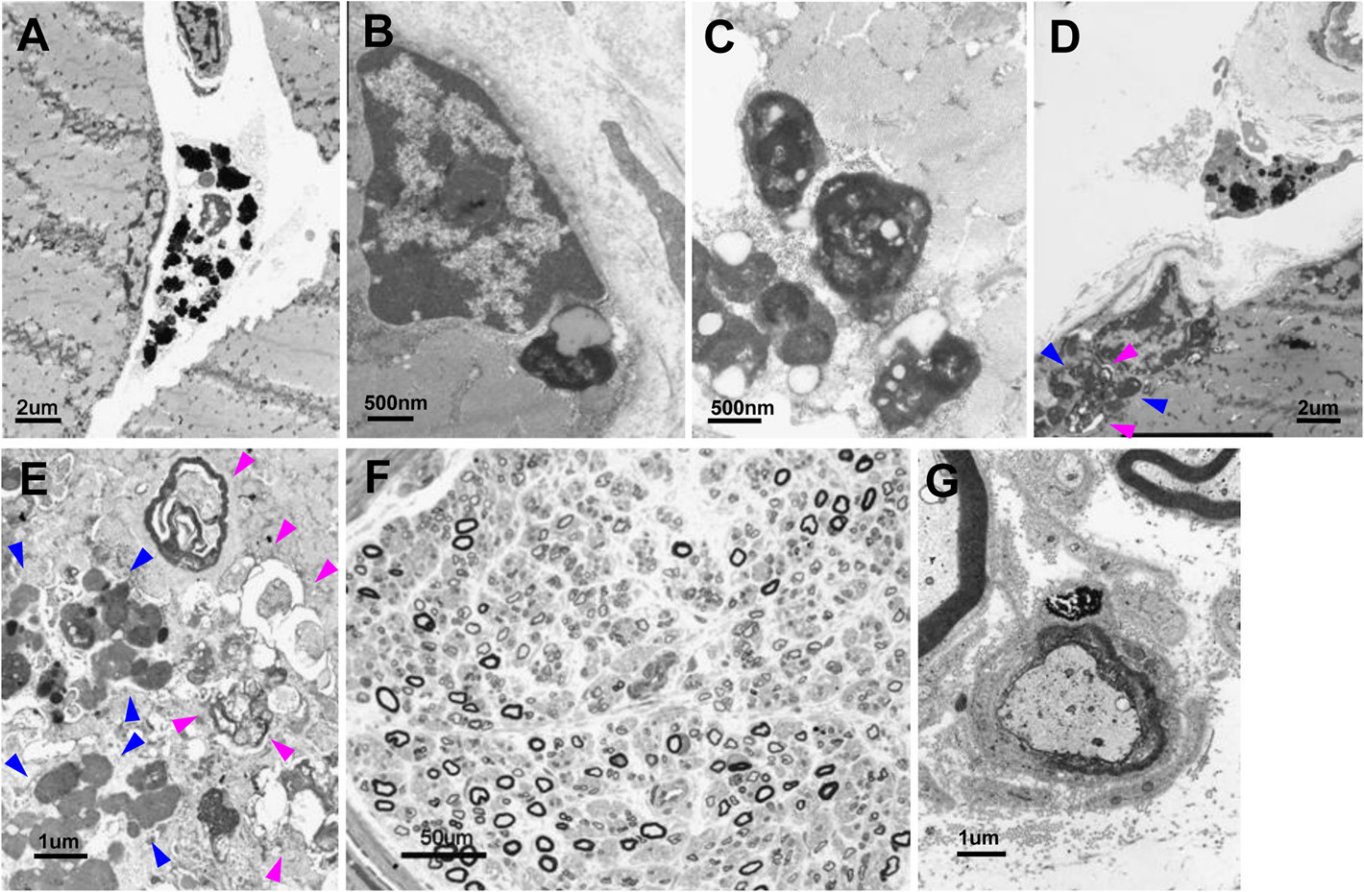
**Electron microscopy of muscle biopsy and nerve pathology. A**: Pigment granules in the cytoplasm of histiocytes. **B**: Isolated pigment granule in the subsarcolemmal cytoplasm. **C**: Pigment granules form a small cluster in a myofiber. **D**, **E**: Autophagic vacuoles (pink arrowhead) in association with numerous pigment granule clusters (blue arrowhead). **F**: Light microscopy of the sural nerve specimen shows a reduction in the number of large-diameter myelinated fibers. **G**: Electron microscopy of the sural nerve specimen shows electron-dense granules in the cytoplasm of a Schwann cell, where the myelin sheath is disrupted.

Electron microscopy was performed on glutaraldehyde-fixed tissue using a standard electron microscopic processing protocol and a transmission electron microscope. Electron microscopy revealed that the pigment granules were localized in the cytoplasm of histiocytes, in the spaces between myofibers, and adjacent to small blood vessels (Figure 3A). In the myofibers, the granules were localized in the subsarcolemmal cytoplasm, either individually (Figure 3B) or in clusters (Figure 3C). Remarkably, autophagic vacuoles were consistently observed in association with many of the granular pigment clusters (Figure 3D, E). Two distinct morphological patterns were evident among the granules found in myofibers: irregularly shaped, highly electron-dense granules, and membrane-bound vesicles of differing electron density with distinct internal structures, often associated with small lipid droplets.

In addition to the standard battery of histological stains, the sural nerve biopsy specimen was embedded in epoxy resin and examined by light and electron microscopy. Light microscopy of the sural nerve showed a reduction in the number of large diameter myelinated fibers, but no other specific features (Figure 3F). However, Prussian blue and Masson Fontana staining revealed perivascular deposits of iron and melanin in the epineurial blood vessels. Electron microscopy revealed a few highly electron-dense granules in the cytoplasm of Schwann cells, where the myelin sheath was disrupted (Figure 3G).

## Discussion

The incidences of minocycline-induced cutaneous pigmentation have been reported in up to 41% of patients with RA who take minocycline [10]. Histochemical staining for pigmented substances (e.g., iron, melanin) has shown that minocycline-induced cutaneous pigmentation can be classified into three or four distinct types [11]. In the present study, histochemical staining of the biopsied muscle demonstrated that the pigments comprised iron and melanin, which is consistent with type II minocycline-induced cutaneous pigmentation [11]. Iron deposition in skeletal muscle has rarely been described in some disorders, including idiopathic hemochromatosis, transfusion induced hemosiderosis, diabetic neuropathy, Waldenstrom’s macroglobulinemia, and AIDS patients [12-14]. Laboratory investigations of our patient showed no evidence of these conditions. No previous study has reported melanin deposition in human skeletal muscle, except for cases of melanoma invasion. Here, we report the first identification of minocycline-induced pigmentation (comprising iron and melanin) in skeletal muscle.

The sural nerve biopsy demonstrated the features of a chronic axonal neuropathy, with minimal pigment observed in the cytoplasm of Schwann cells. Only a few cases of minocycline-associated sensory neuropathy have been reported, and axonal-type sensory neuropathy is a common complication in RA [15]. Thus, it is unclear whether or not the sensory neuropathy observed in our patient is related to minocycline therapy.

**Figure 4:**
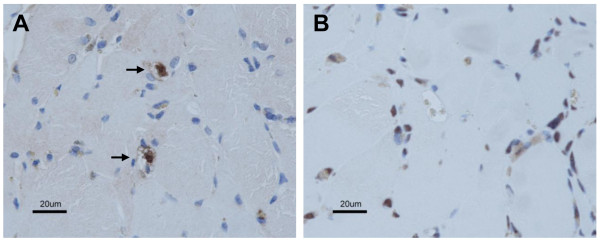
**Immunohistochemistry of muscle biopsy.** Immunohistochemistry was performed on serial paraffin sections. Sections were immersed in citrate buffer, heated in a microwave for antigen retrieval, and immunostained with antibodies against p62/SQSTM1 (1:2000; Progen) (**A**) and TAR-DNA binding protein-43 (1:500; Protein Tech Group) (**B**) according to standard procedures. The immunostaining was visualized with diaminobenzidine tetrahydrochloride and counterstained with methylene blue. Arrow indicates p62 immuno-positive aggregates within rimmed vacuoles. Some nuclei are normally immunostained with TDP-43.

Tissue pigmentation due to minocycline treatment is not usually harmful [9]. However, the striking finding in the biopsied muscle from our patient is the presence of rimmed vacuoles that had acid phosphatase-positive autophagic activity and contained numerous pigmented granules in various forms. The strong association between autophagic vacuoles and the accumulation of minocycline-induced pigments is evident. The rimmed vacuoles were positive for p62/SQSTM1 (p62) immunostaining (Figure 4A), which has been reported as a diagnostic marker for drug-induced AVM [16]. In distal myopathy with rimmed vacuoles (DMRV), activation of autophagy is thought to occur via the accumulation of unfolded/misfolded proteins [2]. No filamentous inclusion (representing the accumulation of unfolded/misfolded proteins) was seen on electron microscopy of samples from our patient. There was also no immunostaining inclusion seen under light microscopy of samples immunostained with an antibody against TAR-DNA binding protein-43 (TDP-43) (Figure 4B). TDP-43-positive inclusions are thought to be a common endpoint of muscle degeneration among the myopathies associated with rimmed vacuoles, such as DMRV, inclusion body myositis and oculopharyngeal muscular dystrophy [17]. All of these conditions have been associated with the presence of filamentous inclusions in myofibers. Thus, our findings suggest that long-term minocycline treatment might cause RVM by activating autophagy through the accumulation of minocycline-induced pigments. Although no previous report has shown minocycline therapy directly linked to autophagy in human disease, two recent studies using different in vitro culture systems found that minocycline induced autophagy and inhibited the growth of tumor cell lines, including glioma cells and epidermoid cancer cells [18,19]. In addition, minocycline-induced pigments are known to form complexes containing minocycline and/or its metabolites [20,21], suggesting that minocycline could directly activate autophagy.

One year after the cessation of minocycline therapy, our patient exhibited no worsening of gait disturbance. There was little improvement in muscle weakness, however, perhaps because she had taken minocycline for an extremely long duration even after the appearance of skin pigmentation. This is an unusual condition. However, long-term minocycline treatment may be used more widely in the future, as on-going clinical trials are assessing the therapeutic use of minocycline as an anti-inflammatory agent in a broad range of disorders. Thus, we must draw attention to this adverse effect because its insidious onset, slowly progressive course, and lack of creatine kinase elevation could complicate its prompt identification in patients.

Chloroquine and hydroxychloroquine are anti-malarial drugs that are also used to treat RA and systemic lupus erythematosus. Hydroxychloroquine is used much more frequently than chloroquine, as the latter is more likely to cause irreversible retinal damage. Long-term hydroxychloroquine treatment rarely causes myopathy and sensory neuropathy; the former typically manifests with an insidious onset and normal-to-mildly-elevated creatine kinase levels, and muscle biopsy consistently reveals curvilinear bodies and muscle fiber atrophy with vacuolar changes [3,22]. After the therapy is discontinued, the resolution of symptoms is slow and may be incomplete. In rare cases, long-term hydroxychloroquine treatment can also cause skin pigmentation [23]. In all reported cases, the main histological feature of hydroxychloroquine-induced pigmentation was the presence of iron and melanin deposits in the dermis [23]. These features are very similar to those observed in our patient, who had never taken the anti-malarial drugs, suggesting that there may be a common pathogenic mechanism underlying these conditions. Hydroxychloroquine myopathy is usually of mild to moderate severity, but a few case of severe hydroxychloroquine myopathy, involving respiratory muscles and/or cardiac myocytes, have been reported [24,25]. However, our patient did not have any symptoms related to respiratory or cardiac dysfunction. Chloroquine and hydroxychloroquine accumulate within lysosomes and are thought to block autophagy by elevating intralysosomal pH and inhibiting lysosomal enzymes [26,27]. However, the exact mechanism underlying myocyte toxicity is unclear. In the future, it will be particularly interesting to examine whether iron and melanin are deposited in the muscles of hydroxychloroquine myopathy patients.

## Conclusions

Minocycline has proven to be an effective disease-modifying anti-rheumatic drug. However, we herein report for the first time that long-term minocycline treatment may cause RVM via the accumulation of minocycline-induced pigmentation in skeletal muscle. The clinical features of minocycline-associated RVM are very similar to those observed in hydroxychloroquine myopathy, suggesting that there may be a common pathogenic mechanism underlying these conditions. As long-term minocycline treatment may be used more widely in the future, we must draw attention to this adverse effect.

### Consent

Written informed consent was obtained from the patient for publication of this case report. A copy of the written consent is available for review by the Editor-In-Chief of this journal.

## Abbreviations

AVM: Autophagic vacuolar myopathy; RVM: Rimmed vacuolar myopathy; RA: Rheumatoid arthritis; p62: p62/SQSTM1; TDP-43: TAR-DNA binding protein-43; DMRV: Distal myopathy with rimmed vacuoles.

## Competing interests

The authors declare that they have no competing interests.

## Authors’ contributions

Study concept and design: KS. Acquisition of the data: KB, ST, KS. Analysis and interpretation: KB, KS, KM, SM, TK. Wrote the paper: KB, KS, SM**.** All authors read and approved the final manuscript.

## Pre-publication history

The pre-publication history for this paper can be accessed here:

http://www.biomedcentral.com/1471-2377/12/140/prepub
